# A Previously Undescribed Helotialean Fungus That Is Superabundant in Soil Under Maritime Antarctic Higher Plants

**DOI:** 10.3389/fmicb.2020.615608

**Published:** 2020-12-18

**Authors:** Kevin K. Newsham, Filipa Cox, Chester J. Sands, Mark H. Garnett, Naresh Magan, Claire A. Horrocks, Jennifer A. J. Dungait, Clare H. Robinson

**Affiliations:** ^1^British Antarctic Survey, Natural Environment Research Council, Cambridge, United Kingdom; ^2^Department of Earth and Environmental Sciences, The University of Manchester, Manchester, United Kingdom; ^3^National Environmental Isotope Facility Radiocarbon Laboratory, Glasgow, United Kingdom; ^4^Applied Mycology Group, Environment and AgriFood Theme, Cranfield University, Cranfield, United Kingdom; ^5^Sustainable Agriculture Sciences, Rothamsted Research, Okehampton, United Kingdom

**Keywords:** Antarctica, ^14^C (or carbon-14), carbon, ^13^C, *Chalara*, Helotiales

## Abstract

We report a previously undescribed member of the Helotiales that is superabundant in soils at two maritime Antarctic islands under Antarctic Hairgrass (*Deschampsia antarctica* Desv.). High throughput sequencing showed that up to 92% of DNA reads, and 68% of RNA reads, in soils from the islands were accounted for by the fungus. Sequencing of the large subunit region of ribosomal (*r*)DNA places the fungus close to the Pezizellaceae, Porodiplodiaceae, and Sclerotiniaceae, with analyses of internal transcribed spacer regions of *r*DNA indicating that it has affinities to previously unnamed soil and root fungi from alpine, cool temperate and Low Arctic regions. The fungus was found to be most frequent in soils containing C aged to 1,000–1,200 years before present. The relative abundances of its DNA and RNA reads were positively associated with soil carbon and nitrogen concentrations and δ^13^C values, with the relative abundance of its DNA being negatively associated with soil pH value. An isolate of the fungus produces flask-shaped phialides with a pronounced venter bearing masses of conidia measuring 4.5–6(7) × 1.8–2.5 μm, suggestive of anamorphic *Chalara*. Enzymatic studies indicate that the isolate strongly synthesizes the extracellular enzyme acid phosphatase, and also exhibits alkaline phosphatase and naphthol-AS-BI-phosphohydrolase activities. Ecophysiological measurements indicate optimal hyphal growth of the isolate at a pH of 4.2–4.5 and a water potential of −0.66 MPa. The isolate is a psychrotroph, exhibiting measureable hyphal growth at −2°C, optimal hyphal extension rate at 15°C and negligible growth at 25°C. It is proposed that the rising temperatures that are predicted to occur in maritime Antarctica later this century will increase the growth rate of the fungus, with the potential loss of ancient C from soils. Analyses using the GlobalFungi Database indicate that the fungus is present in cold, acidic soils on all continents. We advocate further studies to identify whether it is superabundant in soils under *D. antarctica* elsewhere in maritime Antarctica, and for further isolates to be obtained so that the species can be formally described.

## Introduction

Although Antarctica has been neglected in global mass sequencing studies of soil fungi (Tedersoo et al., 2014; Bahram et al., 2018; Egidi et al., 2019; Větrovský et al., 2019), several features are starting to emerge of the fungal communities found in the soils that form on the continent (Bockheim, 2015). One characteristic of Antarctic soil fungal communities is their relatively low alpha diversity (Canini et al., 2020), with reductions in the number of species present in soils at higher southern latitudes (*c.f.* Větrovský et al., 2019), which are associated with decreasing air temperature and, most probably, liquid water availability (Newsham et al., 2016). Perhaps surprisingly for such an isolated continent, another feature of the fungal communities in Antarctic soils is that they exhibit low endemism, with many taxa either having cosmopolitan or bipolar distributions (Cox et al., 2016). Specific fungal taxa are also characteristic of maritime Antarctic soils, with perhaps the foremost example being *Pseudogymnoascus roseus*, which is widespread in the barren fellfield soils of the region (see Supplementary Figure S3 in Newsham et al., 2016). In contrast, fungi in the class Leotiomycetes and the order Helotiales are frequent in the roots of Antarctic Hairgrass (*Deschampsia antarctica*), one of the only two native Antarctic higher plant species, and in the soils that form under the grass (Upson et al., 2009; Cox et al., 2016, 2019).

In the course of studies on maritime Antarctic soils sampled from under *D. antarctica*, a member of the Helotiales was identified that was frequent at Signy Island in the South Orkney Islands and Léonie Island in southern maritime Antarctica (Cox et al., 2016, 2019; Newsham et al., 2018). This fungus, referred to previously as Helotiales sp. 1, respires significant volumes of CO_2_ from ancient (> 1,000 years before present, BP) soil carbon (C) sources (Newsham et al., 2018). Here, we conduct preliminary taxonomic analyses on this fungus by sequencing the large subunit (LSU) and internal transcribed spacer (ITS) regions of its ribosomal (*r*)DNA, describe its anamorph, report its associations with edaphic factors and its enzymatic capabilities, determine its growth responses under controlled conditions to different pH values, soil water potentials and temperatures, and compare these responses with information in the GlobalFungi database (globalfungi.com; Větrovský et al., 2020). In doing so, we build up a picture of how this apparently frequent soil fungus might respond to climatic change in maritime Antarctica (Bracegirdle et al., 2008; Bracegirdle and Stephenson, 2012; Turner et al., 2016), and what the consequences of these responses might be for the soils of the region during the twenty first century.

## Materials and Methods

### Sampling

Soils used in the analyses were sampled in October and November 2011 from active layers overlaying permafrost in six pits (0.40 m × 0.40 m) dug under turves of *D. antarctica*. Three pits were dug at Polynesia Point on Signy Island (60.7107°S, 45.5849°W; altitude 35–45 m a.s.l.) and three were dug on Walton Terraces on the north-western side of Léonie Island (67.5984°S, 68.3561°W; altitude 50–60 m a.s.l.). The three pits dug at each island were separated by a mean distance of 311 m. Soils were sampled by hammering five sterile tubes (50 ml capacity) horizontally into the vertical faces of each pit at depths of 2, 4, and 8 cm, generating 15 soil samples from each pit. Further details of sampling are provided by Cox et al. (2016, 2019) and Newsham et al. (2018). Soil was frozen at −20 or −80°C within a few hours of sampling and was transported back to the United Kingdom at these temperatures, where it was thawed prior to the analyses described below.

### Edaphic Factors

Soil pH, moisture and C and N concentrations were measured in bulked soils from each of the three depths in the three pits dug at each island, generating 18 soil samples in total. Soil pH was measured by adding approximately the same volume of deionized water to each soil sample to generate slurries and recording pH with a glass electrode (pH 21, Hanna Instruments, Leighton Buzzard, United Kingdom). Soil moisture was measured gravimetrically after drying *c*. 1 g of fresh soil at 105°C for 17 h. Sub-samples (*c*. 2.5 mg) of air-dried soil were weighed into foil capsules and were analyzed for total C and N (Carlo Erba NA1500 elemental analyser, CE Instruments, Wigan, United Kingdom). Details of samples are provided by Robinson et al. (2020).

### ^14^C and ^13^C Determinations

Owing to the expense of these determinations, analyses were made on the five replicate soils sampled from depths of 2, 4, and 8 cm in one of the three pits dug at each island, generating 30 samples in total. The soils were submitted to the NERC (now NEIF) Radiocarbon Laboratory, where soil C was converted to carbon dioxide (CO_2_) using either bomb combustion in a high-pressure oxygen atmosphere, or an elemental combustion system (Costech ECS 4010, Italy). Sample CO_2_ was cryogenically purified under vacuum and split into aliquots. The δ^13^C (relative to the Vienna PDB standard) was determined on one aliquot of sample CO_2_ by isotope ratio mass spectrometry (Delta V, Thermo-Fisher, Germany). A second aliquot was converted to graphite using Fe:Zn reduction (Slota et al., 1987) and analyzed for ^14^C content using accelerator mass spectrometry at the Scottish Universities Environmental Research Centre (East Kilbride, United Kingdom). Following convention (Stuiver and Polach, 1977), ^14^C results were normalized to δ^13^C = −25‰ and expressed as % modern and conventional radiocarbon ages (in years BP, where 0 BP = AD1950). Mean residence time (MRT) of C in soil was determined from ^14^C concentration using a bomb-^14^C soil carbon turnover model (the Meathop Model; Harkness et al., 1986), as described by Horrocks et al. (2020). For samples that showed no clear evidence of bomb-^14^C incorporation (i.e., < 97%Modern), the conventional radiocarbon age was used as the MRT (Bol et al., 1999). Sample details, including unique publication codes for each sample, are provided by Robinson et al. (2020).

### DNA and RNA Library Construction

As described by Cox et al. (2016, 2019), both nucleotides were extracted simultaneously from soil samples (5 mg) using RNA PowerSoil Total RNA Isolation and DNA Elution Accessory kits (MoBio Laboratories, Carlsbad, CA, United States). Extracted RNA was treated with a Turbo DNA-free kit (Life Technologies, Carlsbad, CA, United States), checked for contaminant DNA using PCR, and reverse transcribed using AccuScript High-Fidelity Reverse Transcriptase (Agilent, Santa Clara, CA, United States) and random non-amers. Internal transcribed spacer (ITS) regions in the extracted DNA and cDNA were amplified in triplicate PCR reactions using ITS1F (Gardes and Bruns, 1993) and ITS4 (White et al., 1990) primers. The ITS4 primer was modified with the Roche 454 A adapter and a 10 bp barcode specific to each sample, and the ITS1F primer was modified with the 454 B adaptor. The triplicate PCR products were pooled and purified using AMPure XP bead purification (Beckman Coulter, Inc., Brea, CA, United States) and quantified using a Qubit dsDNA HS Assay (Life Technologies, Carlsbad, CA, United States) before normalization to consistent concentration. The purified and normalized PCR products were run on the 454 Roche Titanium FLX platform at the Liverpool Centre for Genomic Research.

The DNA and RNA sequences were processed using the QIIME pipeline (Caporaso et al., 2010). Sequences were filtered to remove reads that were of low quality, < 300 bp or > 1,200 bp, and were split according to barcodes. The remaining sequences were denoised (Reeder and Knight, 2010), and checked for potential chimeras (Edgar et al., 2011). After deleting potential chimeras, the resulting libraries from Signy Island and Léonie Island consisted of 2,313–11,401 and 1,994–15,482 DNA reads and 2,478–11,840 and 5,250–10,943 RNA reads, respectively (Supplementary Figure S1). The ITS2 regions of the remaining sequences were extracted using ITSx (Bengtsson-Palme et al., 2013) and grouped into operational taxonomic units (OTUs) at 97% sequence similarity using USEARCH 6.1 (Edgar, 2010). The ITS sequence of the isolate (see below) showed 100% similarity to a representative OTU in the DNA and RNA libraries, enabling its abundance in field soil to be determined (see Robinson et al., 2020). DNA and RNA sequences have been deposited in the NCBI Sequence Read Archive (accession codes SRP068654 and PRJNA518063, respectively).

### Isolation, Agar Media, and Microscopy

The fungus was isolated in 2012 from surface sterilized organic matter (OM). Briefly, decomposing OM picked from Léonie Island soil was washed 30 times in sterile water, blotted dry on sterile filter paper and pressed into soil extract medium (SEM) before incubation at 7°C for up to 16 weeks (Newsham et al., 2018). An isolate of the fungus was subsequently maintained at *c.* 15°C on half strength potato dextrose agar (PDA) medium. The isolate was also inoculated onto oat agar (OA) and Azure B agar media (Newsham et al., 2018). Phialides and conidia formed on the latter medium at the inoculation point after 6 weeks of growth at *c.* 15°C. Squash mounts in water were examined at ×400 and ×1,000 magnifications using light microscopy (Olympus BX51, Olympus Life Sciences, Tokyo, Japan), with images of structures being captured using Cell P software (Olympus Life Sciences).

### Taxonomic Placement

DNA was extracted from hyphae of the isolate growing on sterile cellophane overlaying half strength PDA medium using a commercially available kit (Extract-n-Amp Plant PCR, Sigma-Aldrich, St Louis, United States). Following standard protocols, the ITS region of *r*DNA was amplified with the ITS1F (5′-CTTGGTCATTTAGAGGAAGTAA-3′)/ITS4 (5′-TCCTCCGCTTATTGATATGC-3′) primer pair, and a portion of the large subunit (LSU) gene was amplified with the JS-1 (5′-CGCTGAACTTAAGCATAT-3′)/LR5 (5′-TCCTGAGGGAAACTTCG-3′) primer pair. The amplicons were bidirectionally sequenced at a commercial facility, using the same primers. The sequences were manually trimmed and consensus sequences were generated prior to deposition in GenBank (accession codes MW033386 and MW033387). The ITS sequences were subjected to blastn searches in UNITE v. 8.2^1^ to determine if the fungus had been sampled and sequenced previously, and to provide a starting point for a taxonomic placement. In order to better place the fungus taxonomically, an exemplar sequence of a voucher LSU sequence was downloaded from NCBI GenBank from each of the classes Orbiliomycetes and Pezizomycetes (as outgroups), and sequences for exemplars from each order within the class Leotiomycetes. Where possible, sequences from type material were chosen to add rigor to the taxonomic placement, with other sequences being derived from material deposited in the Centraalbureau voor Schimmelcultures (CBS) culture collection, the majority of which are reported by Vu et al. (2019), or from vouchered specimens. Sequences were aligned using MAFFT 7.450 (Katoh and Standley, 2013) in the GENEIOUS R11 package (Biomatters, Ltd., Auckland, New Zealand). Ambiguous columns were excised where alignment ambiguities existed around regions containing gaps. A phylogenetic analysis was undertaken to place the test LSU sequence against the voucher sequences, and to identify the likely order to which it belonged. An exemplar sequence from each family in the order was downloaded and a new round of phylogenetic analyses was conducted to place the test LSU sequence to family. An exemplar from each genus of the likely family and closely related families was downloaded to further refine the taxonomic placement. A similar strategy was used to provide context to closely matching ITS sequences in order to produce an ITS phylogeny. Phylogenetic analyses were conducted using approximate maximum likelihood in the package FastTrees 2.1.11 (Price et al., 2010) in the GENEIOUS package using a General Time Reversible (GTR) model, four rate categories per site and the Gamma20 likelihood optimized. The final phylogeny was produced in MrBayes 3.2.3 (Ronquist et al., 2012) using a GTR model with a gamma distribution. The analysis used four heated chains and four replicates. Twenty million generations were run, with sampling every thousandth generation. Convergence, stability and stationarity were determined using Tracer 1.7.1 (Rambaut et al., 2018).

### Responses to pH

The isolate was grown on half strength PDA medium amended to pH values of 3.9, 4.2, 4.5, 5.1, 5.6, and 6.1 with citric acid/phosphate buffer (Stoll and Blanchard, 1990). The pH values of the media were measured with a Jencons pH meter (Jencons Scientific Ltd., Leighton Buzzard, United Kingdom) prior to autoclaving. Media at pH 3.9 and pH 4.2–6.1 were solidified with 2 and 1.5% technical agar (Oxoid no. 1, Thermo Fisher Scientific Ltd., Basingstoke, United Kingdom), respectively. The medium at each pH was poured into six replicate 90 mm diameter non-vented Petri dishes and was inoculated with plugs (7 mm diameter) of the fungus bored from the margins of actively growing colonies on half strength PDA medium. The dishes were incubated at 15°C in the dark for 5 weeks. Two measurements of each colony’s diameter were recorded weekly at *c*. 90° to each other and radial extension rates (mm d^–1^) were subsequently calculated.

### Responses to Temperature

Plugs (7 mm diameter) bored from the margins of the isolate growing on half strength PDA medium were inoculated onto SEM in 0.2 μm-vented sterile cell culture flasks (Thermo Fisher Scientific BioLite, 130189, Loughborough, United Kingdom) that were incubated at −2, 2, 4, 9, 15, 20, and 25°C for up to 5 weeks. Four flasks were prepared for incubation at each temperature. Those at 4 and 15–25°C were incubated in refrigerators or cabinets, the temperatures of which were monitored hourly using TinyTag Plus 2 TGP-4017 loggers (Gemini Data Loggers Ltd., Chichester, United Kingdom). Those at −2, 2, and 9°C were submerged vertically into coolant in recirculating water baths (Newsham and Garstecki, 2007), with all of the flask except the neck being submerged in the coolant. Temperatures were monitored regularly using a thermometer set into SEM in a dummy flask. Measurements of the diameter of each colony recorded at 7 d intervals were subsequently used to calculate radial extension rates (mm d^–1^).

### Responses to Water Potential

Sterile capillary matting (Gardman, Huntingdon, United Kingdom) was placed into 90 mm Petri dishes and was soaked with liquid SEM modified with PEG 8000 to water potentials of −0.66, −0.96, −2.22, −3.15, −4.90, −6.17, and −8.24 MPa, measured using a dew point potentiameter. Sterile black cotton cloth was placed on top of the matting, followed by sterile cellophane film (55 mm × 55 mm; Natureflex 28 NP, Innovia Films, Wigton, United Kingdom). Air bubbles were removed from under the cellophane with a sterile L-shaped spreader. Plugs (7 mm diameter), bored from the margins of colonies grown on SEM for 3 weeks at 15°C, were placed in the centre of the cellophane. The dishes, three of which were prepared for each water potential, were sealed with Parafilm and incubated in the dark at 15°C. Each colony was photographed at weekly intervals for up to 6 weeks. Using ImageJ (National Institute of Health, Bethesda, MD, United States), two measurements of each colony’s diameter were recorded at *c*. 90° to each other. Radial extension rates (mm d^–1^) were subsequently calculated.

### Extracellular Enzyme Analyses

Measurements of extracellular phosphatase activities using *p*-nitrophenol assays were made using the methods described by Newsham et al. (2018), except that the substrate for the assays was 4-nitrophenyl phosphate disodium salt hexahydrate (Sigma-Aldrich, St Louis, United States), with the pH values of the acetate buffer being adjusted to 6.0 and 8.0 for acid and alkaline phosphatase assays, respectively. Semi-quantitative measurements of the activities of these two enzymes were also made at pH 5.4 and pH 8.5 with 2-naphthyl phosphate as the substrate using the methods described by Newsham et al. (2018) and API ZYM kits (bioMérieux Ltd., Basingstoke, United Kingdom). The same methods were also used to semi-quantitatively test for the activities of lipase, trypsin, α-chymotrypsin and α-galactosidase with the substrates 2-naphthyl myristate (pH 7.5), N-benzoyl-DL-arginine-2-naphthylamide (pH 8.5), N-glutaryl-phenylalanine-2-naphthylamide (pH 7.5), and 6-Br-2-naphthyl-αD-galactopyranoside (pH 5.4), respectively.

### Geographical Distribution

The ITS2 region of the fungus was entered into the GlobalFungi database (Větrovský et al., 2020) and the geolocations of exact hits were recorded. After excluding the data reported for Signy Island and Léonie Island reported by Cox et al. (2016), the details of substrates, soil pH, and mean annual temperature and precipitation were noted for the 285 exact hits in the database.

### Statistical Analyses

The effects of soil depth on the relative abundance of the fungus in DNA and RNA communities, and the influence of pH, temperature and water potential on the radial extension rate of the isolate, were tested with ANOVA and Tukey’s multiple range tests. Associations between the relative abundance of the fungus and edaphic factors were tested with linear and three part Gaussian regressions. Analyses were made in Sigmaplot 14 (Systat Software Inc., San Jose, CA, United States) and MINITAB 18 (State College, PA, United States).

## Results

### Relative Abundance in DNA and RNA Libraries

The fungus was present in all 45 soil samples from Signy Island from which DNA libraries were made. Its mean relative abundance in DNA libraries from the island was 39% (range 19–66%; Supplementary Figure S1C). At Léonie Island, it was present in 42 of the 45 soil samples from which DNA libraries were made, with a mean relative abundance of 46% (range 0–92%; Supplementary Figure S1C). RNA of the fungus was detected in 43 out of 45 libraries constructed from Signy Island soil, but at a much lower relative abundance than in DNA libraries (mean relative abundance 2%, range 0–8%; Supplementary Figure S1F), and its RNA was detected in 36 of the 45 RNA libraries from Léonie Island, with a mean relative abundance of 17% (range 0–68%; Supplementary Figure S1F).

### Changes in Relative Abundance With Soil Depth and Soil Fraction

**FIGURE 1:**
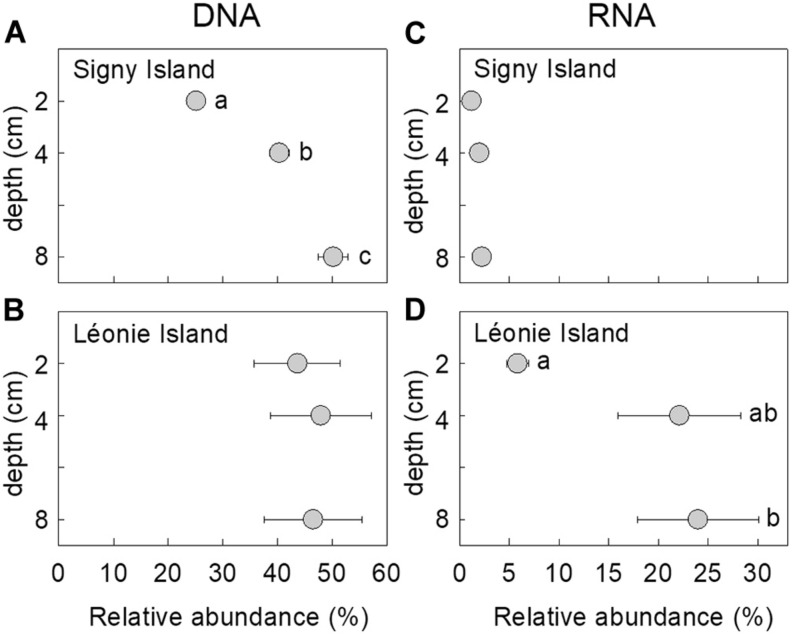
Relative abundances of the fungus in **(A,B)** DNA and **(C,D)** RNA communities as a function of soil depth at Signy Island **(A,C)** and Léonie Island **(B,D)**. Values shown are means ± standard error of the mean. Values marked with different letters differed following Tukey’s tests.

The relative abundance of the DNA of the fungus changed with depth at Signy island, with a doubling in its abundance from 25 to 50% of the DNA community as depth increased from 2 to 8 cm [*F*_*(2, 42)*_ = 37.35, *P* < 0.001; Figure 1A]. There were no apparent effects of soil depth on the relative abundance of the fungus in DNA and RNA communities at Léonie Island and Signy Island, respectively (Figures 1B,C), but its mean abundance in the RNA community at Léonie Island increased from 6 to 24% as depth increased from 2 to 8 cm [*F*_*(2, 42)*_ = 3.87, *P* = 0.029; Figure 1D].

### Associations With Edaphic Factors

**FIGURE 2:**
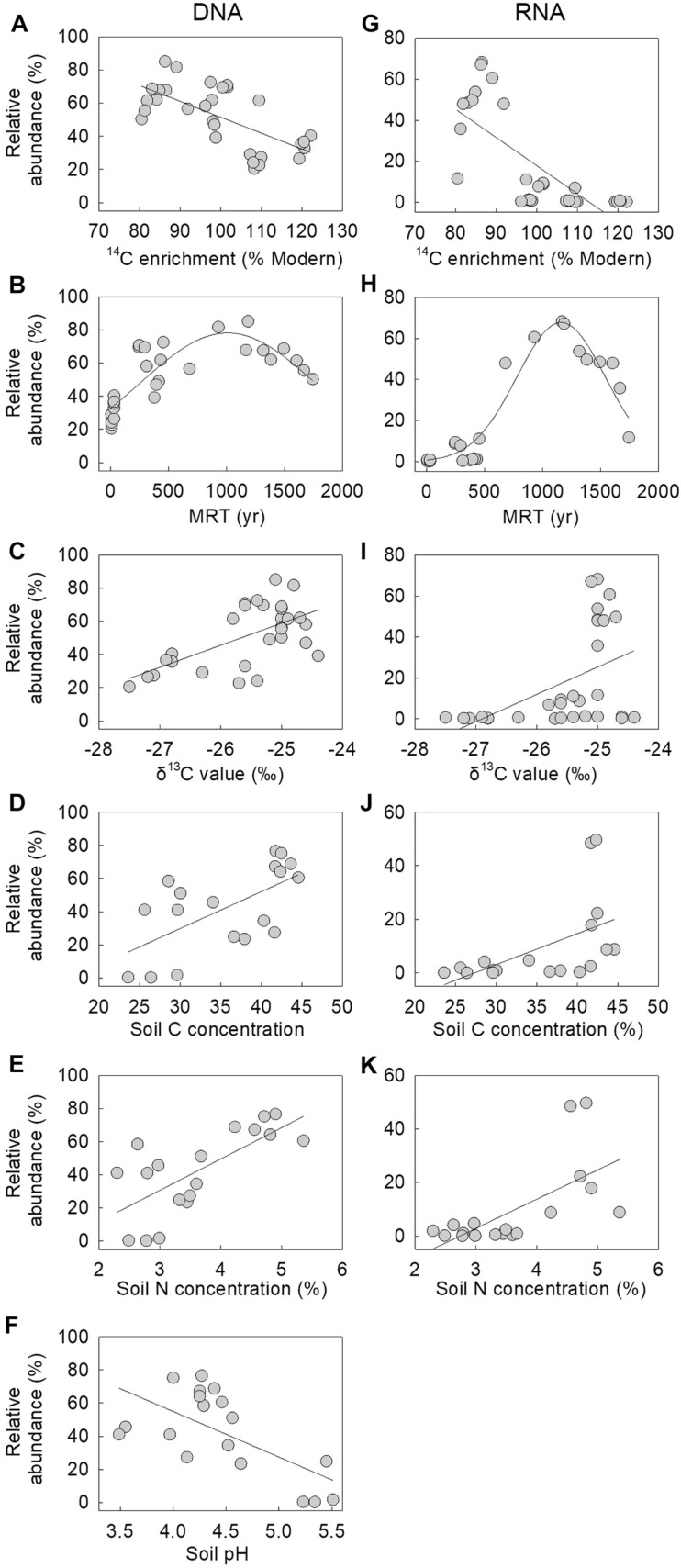
Relative abundances of the fungus in **(A–F)** DNA and **(G–K)** RNA communities as a function of soil ^14^C enrichment, mean residence time (MRT), δ^13^C content, C and N concentrations and pH value. Lines are best fits, derived from linear and **(B,H)** three-parameter Gaussian functions. Note that one sample, assigned a MRT of 9 or 77 years, was excluded from **(B)** and **(H)**, and that the association between the relative abundance of the fungus in the RNA community and soil pH value was not significant.

Correlative analyses between the relative abundance of the fungus in the DNA and RNA communities at Signy Island and Léonie Island indicated that it was more frequent and active in soils with lower radiocarbon (^14^C) enrichment (Figures 2A,G), with significant negative linear associations between its abundance in the two communities and ^14^C enrichment (*r* = −0.670 and −0.743, both *P* < 0.001). There were also significant associations between the MRT of soil C and the relative abundance of the DNA and RNA of the fungus, with peak abundance of each nucleotide in soils aged approximately 1,000 and 1,200 years BP, respectively (*r* = 0.835 and 0.965, both *P* < 0.001; Figures 2B,H). The relative abundances of the DNA and RNA of the fungus were positively and linearly associated with soil δ^13^C value and C and N concentrations (*r* = 0.525–0.699, all *P* < 0.025; Figures 2C–E,I–K), whereas the abundance of its DNA was negatively associated with soil pH value (*r* = −0.646, *P* = 0.004; Figure 2F). Soil moisture concentration, which ranged from 58.4 to 76.2%, was not associated with the relative abundance of the fungus in the DNA or RNA communities (data not shown).

### Taxonomic Placement

**FIGURE 3:**
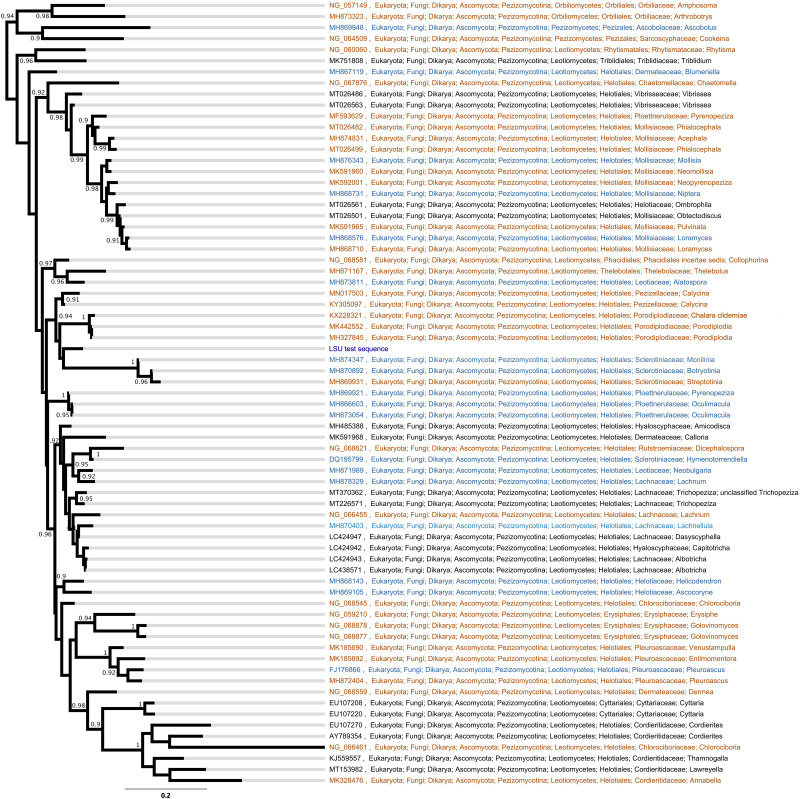
Bayesian phylogeny of large ribosomal subunit (LSU) sequences from type material (orange), CBS cultures (blue), and vouchered specimens (black). Node support numbers are posterior probabilities. Tip labels are GenBank accession codes and the taxonomic cascade is derived from NCBI GenBank.

The LSU phylogeny placed the fungus in the order Helotiales. Type, CBS and voucher LSU sequences were able to refine the test sequence to matching to members of the families Pezizellaceae, Porodiplodiaceae, and Sclerotiniaceae, such as *Chalara clidemiae* and species of *Calycina*, *Porodiplodia*, *Monilinia*, *Botryotinia*, and *Streptotinia* (Figure 3), but with low support (posterior probability of 0.94) and without sufficient taxonomic coverage of genera available to gain any more precision. The ITS phylogeny similarly placed the fungus in the Helotiales, and indicated close matches to sequences assigned to the families Pezizellaceae, Hyaloscyphaceae, and Dermateaceae (Supplementary Figure S2). Comparisons of the ITS region sequence of the fungus with those in the UNITE database indicated that it bore close (> 99%) similarities to soil and root fungi from cold regions (Table 1). The best match was to the sequence of a fungus cloned from acidic (pH 4.4) soil under *Eriophorum*, *Betula*, *Ledum*, and *Vaccinium* spp. at Toolik Lake in the Low Arctic (Table 1). Other close matches were recorded with a *Phialea* species inhabiting the hair roots of the ericaceous plant species *Phyllodoce aleutica* growing in an alpine region of Japan, and members of the Helotiales in soil under the shrub *Salix polaris* growing at Abisko in sub-Arctic Sweden, in *Poa flabellata* roots from the Falkland Islands, and a member of the Helotiaceae from the roots of *Tsuga canadensis* growing in an alpine region of North America (Table 1).

**TABLE 1:** The 10 closest matches to the ITS sequence of the fungus derived from UNITE v. 8.2 (accessed August 2020), showing accession codes, UNITE taxon names, similarity scores and percentage similarities and regions and substrates of origin.

Accession code*	UNITE taxon name	Score	Coverage (%)	Similarity (%)	Region of origin	Substrate	References
HQ211994	Helotiales	983	89	99.813	Low Arctic (Alaska)	Soil	Deslippe et al., 2012
HQ211950	Helotiales	983	89	99.813	Low Arctic (Alaska)	Soil	Deslippe et al., 2012
UDB0756281	Envir: Fungi	983	51	99.813	United States	Soil	Tedersoo et al., 2014
LC131017	*Phialea*	981	99	99.813	Tateyama Kurobe Alpine Route (Japan)	*Phyllodoce aleutica* hair roots	Unpublished
EU529971	Helotiales	981	100	99.813	Sub-Arctic (Abisko, Sweden)	Soil under *Salix polaris*	Hrynkiewicz et al., 2009
HQ212087	Helotiales	977	89	99.626	Low Arctic (Alaska)	Soil	Deslippe et al., 2012
MN328304	Helotiales	974	99	99.811	Cool southern temperate (Falkland Islands)	*Poa flabellata* root	Unpublished
MN328301	Helotiales	974	99	99.811	Cool southern temperate (Falkland Islands)	*P. flabellata* root	Unpublished
MN328296	Helotiales	974	99	99.811	Cool southern temperate (Falkland Islands)	*P. flabellata* root	Unpublished
KF359562	Helotiaceae	972	100	99.440	Great Smoky Mountains (United States)	*Tsuga canadensis* root	Baird et al., 2014

*Note that UDB0756281 is deposited in UNITE. All other sequences are deposited in GenBank.

### Descriptions of Colonies and Anamorph

**FIGURE 4:**
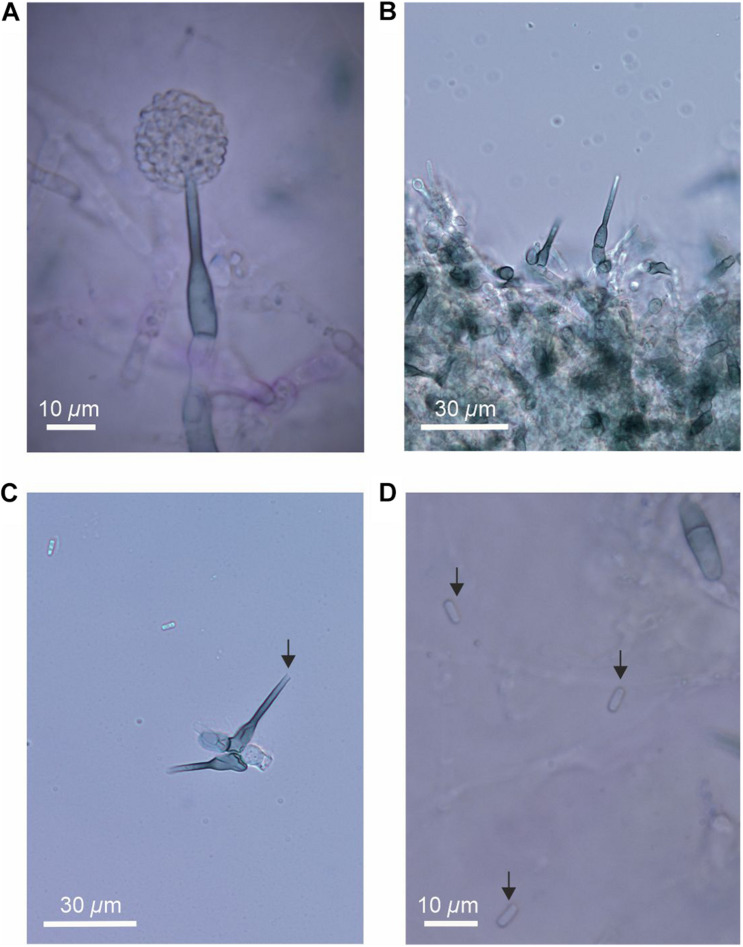
The anamorph of the fungus, showing **(A)** flask-shaped phialide with a pronounced venter bearing masses of conidia, **(B)** phialides and swollen cells, **(C)** phialide without flared apex (arrowed), and **(D)** conidia (arrowed).

On half strength PDA and OA medium, colonies of the fungus are thin and white with well-defined margins. Sclerotia and stroma are absent. Conidia are produced in masses at the apices of pigmented, flask-shaped phialides of length 25–37 μm and widths 5–7 μm (venter) and 2.0–2.5 μm (collarette; Figures 4A,B). The apices of phialides are not flared (Figure 4C). Conidia, which were not observed to form in basipetal chains, are hyaline, cylindrical with rounded ends, 0(–1) septate and measure 4.5–6(7) × 1.8–2.5 μm (Figure 4D).

### Responses to pH, Temperature, and Water Potential

**FIGURE 5:**
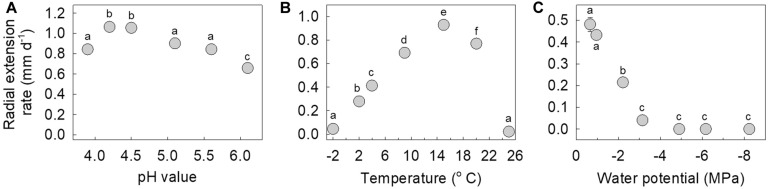
Radial extension rate of the fungus in response to **(A)** pH values between 3.9 and 6.1, **(B)** temperatures between –2 and 25°C, and **(C)** water potentials between –0.66 and –8.24 MPa. Note that values are means of 3–6 replicates ± standard error of the mean, with all but one of the error bars being obscured by the data points. Distinct letters within each panel denote values that differed significantly following Tukey’s tests.

The isolate is an acidophile, exhibiting maximum hyphal growth at pH 4.2–4.5, and slower growth at pH 6.1 compared with pH 3.9–5.6 (Figure 5A). It was also found to be a psychrotroph, with measurable hyphal growth at −2°C (0.04 mm d^–1^) and optimum hyphal extension rate at 15°C (0.93 mm d^–1^). Radial extension rate at 25°C was negligible (0.02 mm d^–1^; Figure 5B). Measurements of hyphal growth on SEM modified with PEG 8000 to a range of water potentials indicated that radial extension rate was fastest at −0.66 MPa, with rapidly declining extension rates between −0.66 and −3.15 MPa, and no growth at below −4.9 MPa (Figure 5C).

### Enzyme Analyses

Analyses using *p*-nitrophenyl assays indicated mean acid phosphatase activity of the isolate of 12 μM h^–1^ *p*-nitrophenol liberated, with no apparent alkaline phosphatase activity (data not shown). API ZYM assays indicated strong acid phosphatase activity, and weak alkaline phosphatase activity. These assays also showed weak naphthol-AS-BI-phosphohydrolase activity, and no activities for lipase, trypsin, α-chymotrypsin and α-galactosidase (data not shown).

### Geographical Distribution

**FIGURE 6:**
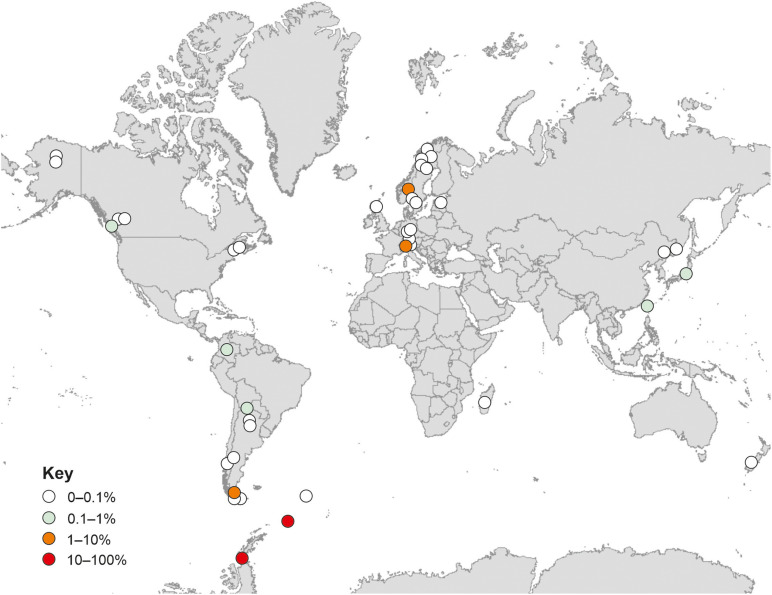
Global distribution of the fungus. Data are from GlobalFungi.com (Větrovský et al., 2020), accessed in September 2020. Colours denote different relative abundances in high throughput sequencing libraries (see key).

Analyses using the GlobalFungi Database indicated that fungi with ITS2 regions bearing 100% similarities to that of the fungus reported here are present on all continents (Figure 6). These analyses also indicated increased relative abundances (> 1%) of the fungus at high latitudes in the southern hemisphere and alpine regions in the northern hemisphere (Figure 6). DNA of the fungus has mainly been amplified from soils in forest, desert, tundra and grassland biomes (91.6% of records), with occasional records from litter, bryophyte tissues and roots. The locations in which the fungus has been recorded have a mean annual temperature of 5.0°C (range −10.4–17.7°C) and a mean annual precipitation of 1,077 mm per annum (range 206–4,251 mm). The mean pH value of the soils from which the DNA of the fungus has been amplified is 4.8 (range 2.6–6.1).

## Discussion

The observations reported here indicate the superabundance of a fungus in two maritime Antarctic soils under *Deschampsia antarctica*. The fungus was recorded in all DNA libraries constructed from soil sampled from Signy Island and all but three of the 45 DNA libraries made from Léonie Island soil, with maximum abundances in the soils from each island of 66 and 92%, respectively. It also occurred in 36–43 of the RNA libraries constructed from each island, but, as noted previously by Cox et al. (2019), its relative abundance in the RNA community (0–68%) was lower than in the DNA community. Although the abundance of the fungus in DNA and RNA libraries may be owing to its nucleic acids being more resistant to damage than those of other fungal taxa (Willerslev et al., 2004), it exhibits similar features to those of other abundant soil fungi. In a recent analysis, it was shown that just 83 fungal taxa, all but three of which are ascomycetes, account for about one fifth of DNA reads from soils sampled from six other continents (Egidi et al., 2019). These fungal taxa, five of which belong to the Helotiales, are characterized by the expression of genes for stress tolerance, CAZymes (carbohydrate active enzymes) and competitive ability (Egidi et al., 2019). Although the competitive abilities of the fungus reported here have yet to be tested, it strongly synthesizes extracellular enzymes for carbohydrate breakdown (Newsham et al., 2018), and it is abundant and active in soils that are frozen for *c*. 8 months per annum (Convey et al., 2018) and which are exposed to annual temperature ranges of 48°C and midwinter temperatures below −20°C (Bokhorst et al., 2011), suggesting that it expresses stress tolerance genes. We hence propose that a combination of these characteristics enable the survival of the fungus in the harsh environments of maritime Antarctica, leading to its superabundance in the soils of Signy and Léonie islands.

In agreement with previous studies showing significant effects of depth on the frequencies of distinct fungal guilds and taxa in soil (Dickie et al., 2002; Lindahl et al., 2006), the fungus reported here was more abundant and active in deeper soils, with, as depth increased from 2 to 8 cm, a doubling in its relative abundance in the DNA community at Signy Island, and a threefold increase in its abundance in the RNA community at Léonie Island. The preference of the fungus for deeper, and hence older (Horrocks et al., 2020), soils resulted in its relative abundance in both nucleic acid communities being negatively associated with soil ^14^C enrichment, with peak abundances of DNA and RNA reads in soils with MRT of C of 1,000–1,200 years BP. The increased relative abundance of the fungus in soils with enriched ^13^C values, derived from plants growing under wetter conditions (Wasley et al., 2006), also agrees with its preference for growth at increased water availability, with maximum hyphal extension rate being recorded here at a water potential of −0.66 MPa. However, we cannot discount the possibility that the positive association between the relative abundances of the fungus in DNA and RNA communities and soil ^13^C value may have arisen from ^13^C enrichment at increased soil depth (Horrocks et al., 2020), possibly arising from isotopic fractionation or the addition of ^13^C-enriched microbially-derived C during decomposition (Schweizer et al., 1999; Boström et al., 2007), or from the depletion of the ^13^C content of plant material entering soil over the previous 150 years (Leavitt and Lara, 1994; Royles et al., 2013).

A previous biogeographical study concluded that the fungal taxa encountered in maritime Antarctic soils tend not be endemic to the region, but instead usually have bipolar or cosmopolitan distributions (Cox et al., 2016). In support of this, analyses using the GlobalFungi Database (Větrovský et al., 2020) confirmed that the fungus studied here is apparently globally distributed, with exact matches of its ITS2 region to DNA sequences amplified from soils on all continents. Excluding the data reported here, further analyses using the GlobalFungi Database indicated that the fungus is most frequent in soils with a mean pH value of 4.8, and is apparently not present in soils with pH values of > 6.2. These findings closely corroborate the analyses here, with the relative abundance of the DNA of the fungus being negatively associated with soil pH, and with the isolate of the fungus exhibiting optimum hyphal extension rate at pH 4.2–4.5 and a marked reduction in extension rate at pH 6.1. As well as being an acidophile, the isolate is a psychrotroph, exhibiting measurable growth at −2°C, optimum hyphal extension rate at 15°C, and negligible growth at 25°C. These findings corroborate analyses using both the GlobalFungi and UNITE databases, which indicate that the fungus tends to occur in cold habitats with a MAT of 5°C, and that its closest matches are found in cold regions, with 99.4–99.8% similarity of its ITS region to members of the Helotiales in soils and roots in the Low Arctic, alpine regions and the cool temperate Falkland Islands (Hrynkiewicz et al., 2009; Deslippe et al., 2012; Baird et al., 2014). These analyses were also in agreement with the wider ITS phylogeny, which showed a distinctive clade formed by the test sequence and its closest matches with no clear generic or familial alignment, suggestive of an as yet undescribed taxon group encompassing cold tolerant fungi.

Phylogenetic analyses of the LSU region of *r*DNA of the isolate provided better resolution at the family level, with the test sequence grouping, albeit at low support, with members of the Sclerotiniaceae, Pezizellaceae and the recently recognized family Porodiplodiaceae (Crous et al., 2018). Morphological characters suggest that membership of the Sclerotiniaceae is unlikely, with an absence of sclerotia and stroma in culture (Carbone and Kohn, 1993). However, the morphology of the phialides is suggestive of *Chalara*, members of which produce flask-shaped, obclavate phialides with pronounced venters (Nag Raj and Kendrick, 1975) and are frequent in the Pezizellaceae. We note that the isolate is dissimilar to *Chalara antarctica*, a member of the family isolated from soils under *D. antarctica* and *Colobanthus quitensis* on the Danco Coast, which has floccose or tufted brown colonies with slender phialides of length 25–30 μm and width 3 μm that lack a pronounced venter, and has a flared collar bearing 2–3 conidia borne in chains (Cabello, 1989). It does, however, bear similarities to the *Chalara* anamorph of *Cyathicula strobilina*, recorded on decomposing cones of *Picea abies* from northern Europe, the colonies of which, as here, display optimal hyphal extension at 10–20°C, but minimal growth at 24°C (Gams and Philippi, 1992). Although dissimilar to *Porodiplodia*, the type genus in the Porodiplodiaceae, colonies of which are darkly pigmented on PDA and OA and produce broad, pigmented, 1-septate conidia in short chains (Crous et al., 2018, 2019), the isolate bears morphological similarities to *C. clidemiae*, another member of this family (Crous et al., 2016). In addition, it has an LSU region sequence with 94% pairwise identity (sequence length 807, identical bases 756) to that of the holotype of *C. clidemiae* (GenBank accession code KX228321; Crous et al., 2016). However, despite these similarities to *Chalara*, given the low levels of support for placement of the isolate at the family level, it is apparent that further research is needed to accurately determine its taxonomic position.

In the latter half of the twentieth century, surface air temperatures in maritime Antarctica rose by *c.* 0.7–2.8°C (Adams et al., 2009), with widespread effects in the environment, such as glacial recession (Cook et al., 2005), ice shelf disintegration (Cook and Vaughan, 2010) and the expansion of populations of vascular plant species, notably *D. antarctica* (Fowbert and Smith, 1994; Gerighausen et al., 2003). Although this warming trend has slowed in the last two decades (Turner et al., 2016), further increases in atmospheric greenhouse gas concentrations are predicted to lead to 2–4°C rises in air temperature by the end of the twenty first century (Bracegirdle et al., 2008; Bracegirdle and Stephenson, 2012), with consequent rises in surface soil temperatures (Qian et al., 2011). Given the superabundance of the fungus reported here in soils at two maritime Antarctic islands, the question arises of what the effects will be of these changes in soil temperature on its growth and activity. It seems likely, given the positive growth response of the fungus to increasing temperatures between −2 and 15°C, and with mean summertime soil surface temperatures on Signy Island and Anchorage Island, a member of the Léonie Islands group, of 2.2–4.4°C (Bokhorst et al., 2008), that warming will lead to increased hyphal growth of the fungus in soil during summer. With soil surface temperatures at Signy Island and Léonie Island reaching 21.2–27.4°C under cloudless skies during austral summer (Bokhorst et al., 2011), it is also possible that the hyphal growth of the fungus, which exhibits marked reductions at > 20°C, may be inhibited. However, we do not anticipate substantial inhibitory effects on its growth rate, since the fungus is most frequent in maritime Antarctic soils at depths of at least 8 cm, which do not heat to the same extent as surface soils (Bokhorst et al., 2013).

What are the likely effects of increased growth and activity of the fungus on the cycling of elements in maritime Antarctic soils? Assuming that the water potential of these soils does not fall below −4.9 MPa—at which point hyphal growth halts—it is likely that C cycling will be accelerated, since the fungus strongly synthesizes extracellular cellulase enzymes (Newsham et al., 2018). With the analyses here showing that the fungus is most frequent in soils containing C with a MRT of 1,000–1,200 years BP, along with data from *in vitro* experiments showing that it respires CO_2_ from Léonie Island soil aged up to *c.* 1,171 years BP (Newsham et al., 2018), it is reasonable to conclude that as soils warm, increased hyphal growth will lead to the loss of ancient C, potentially aged up to 5,500 years BP (Björck et al., 1991). In agreement with previous studies on other Ascomycetes (e.g., Oses-Pedraza et al., 2020), effects of the fungus on the sparse lignin moieties present in maritime Antarctic soils are unlikely, as it displays no apparent activity of peroxidase-type lignin modifying enzymes (Newsham et al., 2018). Data from the present study and that of Newsham et al. (2018) also indicate few effects of the fungus on the breakdown of glycoproteins, glycolipids, esters, proteins, polypeptides or amino acids, with the fungus exhibiting minimal or no activity of esterase, esterase lipase, α-galactosidase, trypsin, α-chymotrypsin or cysteine or valine arylamidases. Nevertheless, as reported here, the fungus strongly synthesizes acid phosphatase and weakly synthesizes alkaline phosphatase and naphthol-AS-BI-phosphohydrolase, with potential effects on the cycling of phosphorus (P) in soils under *D. antarctica* as they warm in future decades. However, with comparatively high extractable concentrations of P in soil under the grass on Signy Island (50–130 mg P kg^–1^ soil; Allen et al., 1967), it is possible that these effects will not influence P cycling to the extent that primary productivity is affected.

## Conclusion

We report a previously undescribed member of the Helotiales that is superabundant in soils under *Deschampsia antarctica* at two maritime Antarctic islands. The relative abundances of its DNA and RNA reads were closely associated with ^14^C enrichment and MRT of soil C, which, together with strong cellulase activity (Newsham et al., 2018), imply significant loss of ancient C from soils as the maritime Antarctic warms over future decades. Phylogenetic analyses based on the LSU region of *r*DNA indicate its placement in, or close to, the families Porodiplodiaceae, Sclerotiniaceae, and Pezizellaceae, with similarities to anamorphic *Chalara*. We advocate that further isolates of the fungus should be obtained prior to the formal description of what appears to be a previously undescribed, globally distributed soil fungus.

## Data Availability Statement

The data are reported by Robinson et al. (2020) at this link: https://doi.org/10.5285/65359151-158C-47D1-8C04-A59BC3A28F53.

## Author Contributions

CR, KN, and JD secured the funding. KN, CR, and FC conducted the fieldwork. FC created DNA and RNA libraries and, with KN, isolated the fungus. CS performed the phylogenetic analyses. MG made ^14^C and ^13^C determinations. CH measured soil C and N concentrations. KN and NM conducted the ecophysiological studies. KN wrote the manuscript, which was commented on and improved by the other authors. All authors contributed to the article and approved the submitted version.

## Conflict of Interest

The authors declare that the research was conducted in the absence of any commercial or financial relationships that could be construed as a potential conflict of interest.

## Footnotes

1 https://unite.ut.ee/
