# Supplementary figures and images for: A Previously Undescribed Helotialean Fungus That Is Superabundant in Soil Under Maritime Antarctic Higher Plants
Source: Front Microbiol. 2020 Dec 18;11:615608. doi: 10.3389/fmicb.2020.615608 (PMC7775421; doi:10.3389/fmicb.2020.615608)

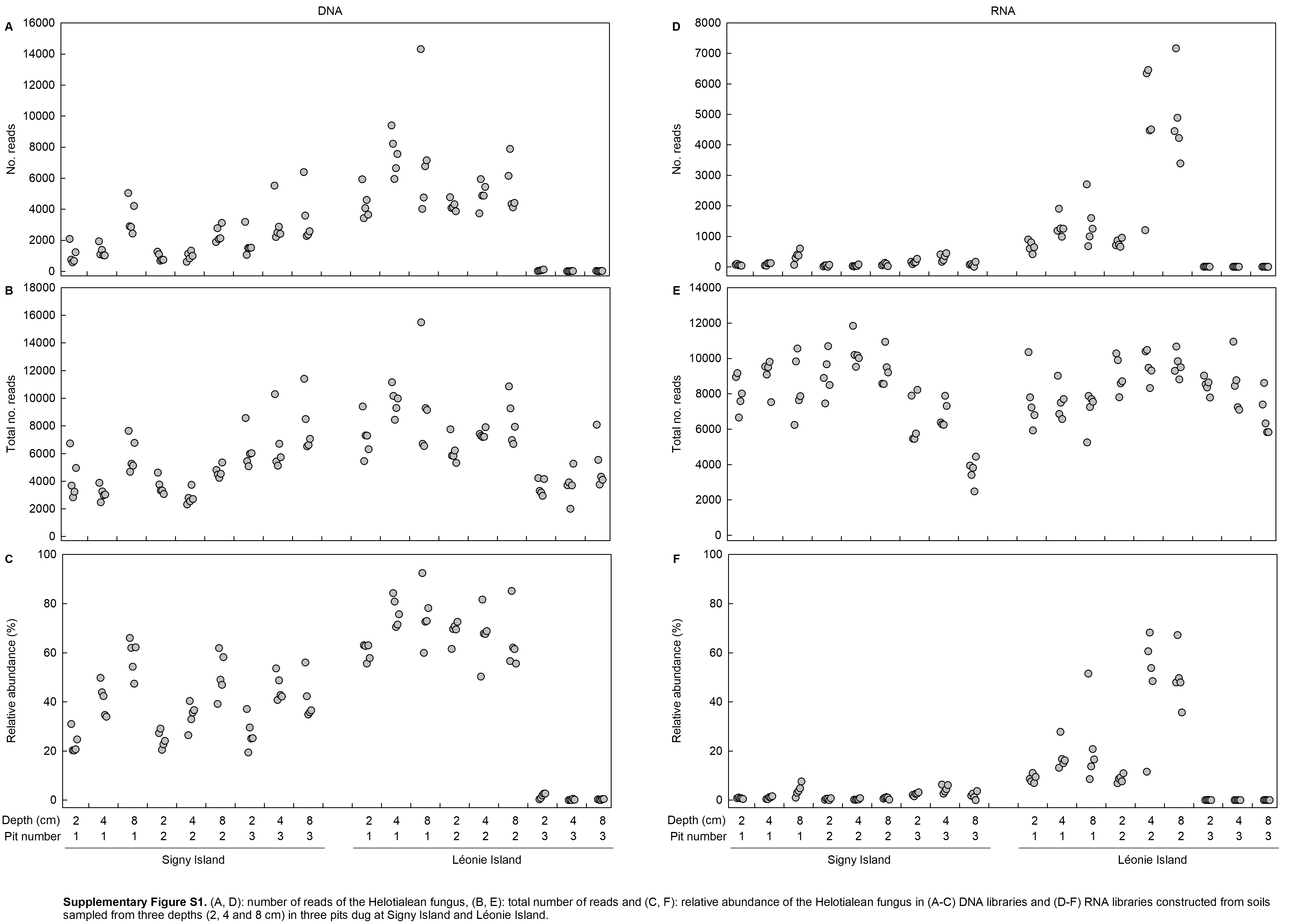

Supplement: Supplementary file 1 [file Image_1.JPEG]

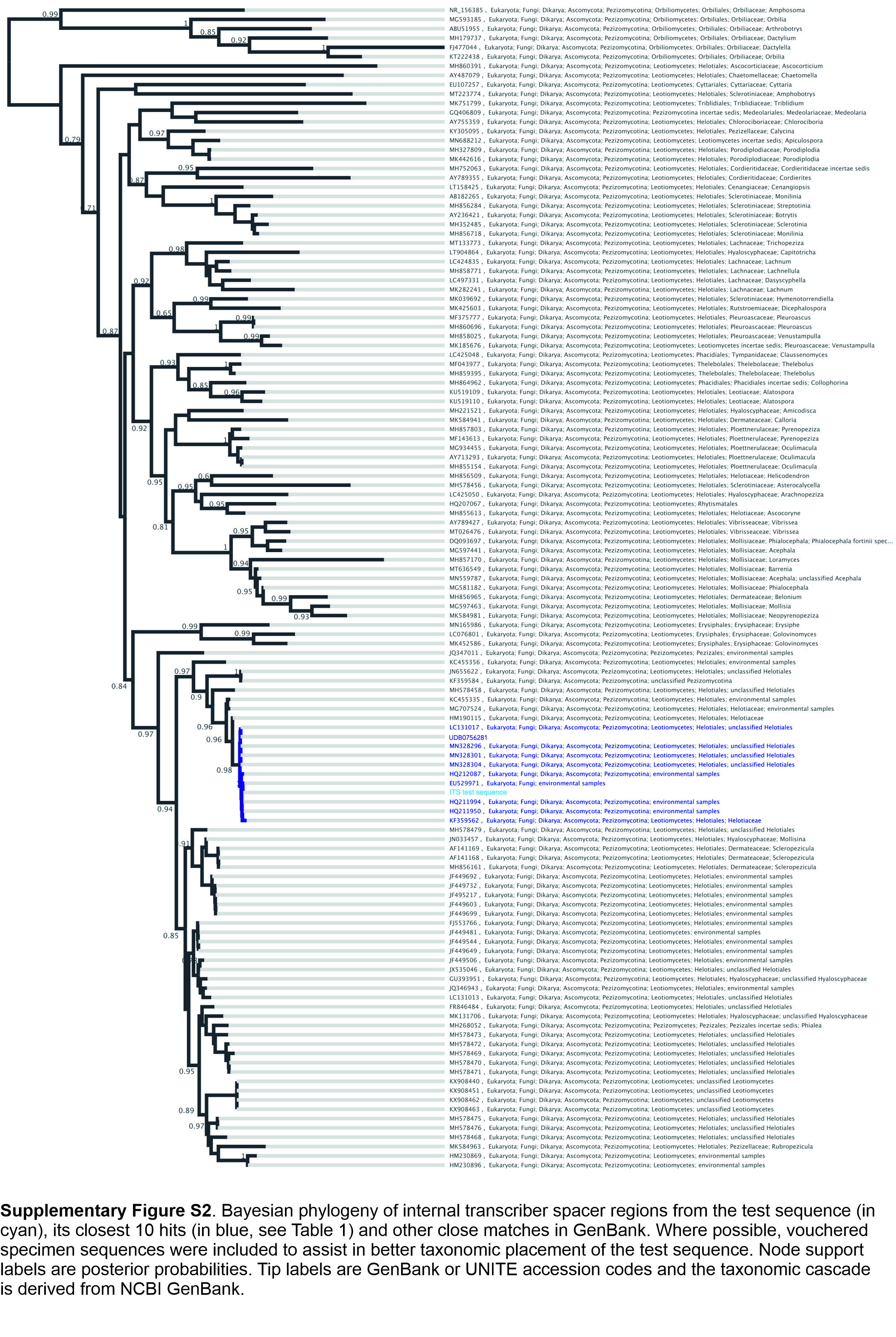

Supplement: Supplementary file 2 [file Image_2.JPEG]
